# Life review in advanced age: qualitative research on the ‘start in life’ of 90-year-olds in the Lothian Birth Cohort 1921

**DOI:** 10.1186/s12877-016-0246-x

**Published:** 2016-04-01

**Authors:** Hilary Lapsley, Alison Pattie, John M. Starr, Ian J. Deary

**Affiliations:** Department of General Practice and Primary Health Care, University of Auckland, Private Bag 92019, Auckland, 1142 New Zealand; Centre for Cognitive Ageing and Cognitive Epidemiology, Department of Psychology, University of Edinburgh, 7 George Square, Edinburgh, EH8 9JZ Scotland UK

**Keywords:** Ageing, Advanced age, Early experience, Qualitative, Life review, Longitudinal

## Abstract

**Background:**

This research report presents findings on ‘start in life’ from a qualitative study of 90-year-olds from the Lothian Birth Cohort 1921. The study aimed to contextualise the LBC1921 cohort in time and place, describe cohort members’ experiences of family and schooling and stimulate further inquiry into the relationships between ‘start in life’ and risk and resilience factors relating to longevity and healthy ageing. Scottish education and family life in the early 1930s are briefly described.

**Methods:**

**Life review questionnaire:** A qualitative Life Review Questionnaire was developed, requiring free-text handwritten responses. Its ‘Start in Life’ section focused on schooling and family support.

**Sample:** Wave 4 of the Lothian Birth Cohort 1921 involved testing 129 members near to their 90^th^ birthday. They reside largely in Edinburgh and its environs. The Life Review Questionnaire was administered to 126 participants, 54 % women.

**Qualitative analysis:** Thematic analysis was the qualitative technique used to categorise, code and extract meaning from questionnaire text. Narratives were extracted from the data to present illustrative stories.

**Results:**

Narratives of start in life gave contextual description. Thematic analysis showed LBC1921 members enjoying their schooling, highlighting teachers, academic achievement, school activities and school friendships. Personal qualities, family circumstances and aspects of schooling sometimes hindered educational performance. Family life was recalled mostly with warmth and parents were often portrayed as valuing education and supporting learning and development. Family adversity from poverty, parental illness and parental death was often mitigated by support from parents (or the remaining parent). Overall, most cohort members believed that they had got off to a good ‘start in life’.

**Conclusions:**

This qualitative investigation of ‘start in life’ adds context and richness to quantitative investigations of the sizeable LBC1921 cohort, stimulating fresh insights and hypotheses into the relationship between child risk and resilience factors that may influence ageing. It demonstrates the utility and wider application of the Life Review Questionnaire. Although the surviving cohort is not representative of their childhood peers, their words provide insight into the processes of weaving experience and memory into a rich texture of meanings that may help create wellbeing across a lifetime.

## Background

Longitudinal and life course studies have contributed significantly to the vast research literature demonstrating that favourable early life circumstances strongly contribute to adult health, resilience, wellbeing, social status and longevity. Unfavourable circumstances, including poverty, malnutrition, abuse, adverse social positioning and fewer years in education have been shown to be risk factors that have an enduring and negative impact on adult lives [1, 2]. As cohorts of established longitudinal studies mature, research attention increasingly turns to the impact of early life on late adulthood and the older years and evidence is accumulating that childhood exposures to adverse situations have a long reach [2]. There is also interest in how adult experience might moderate childhood adversity and its potential impact on old age [3]. Conceptualising just how early experience makes its mark is not easy, however. Theories differentiate between direct impact, with its effects on growth and development, and indirect impact, mediated by later circumstances (for example, lower socioeconomic status (SES) in childhood is associated with lower SES in adulthood, which can, in itself, lead to poor health outcomes) [4–6]. The concept of functional reserve is also an important theoretical contribution, suggesting that optimal development during childhood can contribute resources to be drawn on in later life [7].

Early circumstances shape us, but so do individual talents and other characteristics. The long-term impact of individual differences across the life course is also a lively field of investigation, with studies from the Lothian Birth Cohort 1921 making substantial contributions, especially regarding cognitive differences. The Lothian Birth Cohort 1921 (LBC1921) is a group of people born in 1921, mainly from Edinburgh and its environs, most of whom at age ~11 in 1932 took a test of mental ability as part of a national intelligence testing survey across Scotland [8, 9]. First contacted at the average age of 79, participants have contributed to an extensive LBC1921 database comprising five waves of assessment that collected cognitive, other psychological, social and health data. LBC1921 is unique internationally in supplying measures of individuals’ intelligence over a time span of more than 80 years and has “offered a rare opportunity to examine the distribution and causes of cognitive ageing across most of the human life course” [10]. Studies of the LBC1921 cohort (and the younger LBC1936 [10]) have so far given rise to many novel findings about the causes of cognitive ageing differences, how cognition changes over time, how individual differences impact on the life course and how cognitive ageing is related to physical health, longevity and other factors [11]. This research has shown that individual differences in intelligence are substantially stable across a lifetime, that intelligence at age 11 predicts longevity and that contributors to later intelligence include education, occupation, health, genetic factors, and brain structure [10, 12, 13].

Qualitative studies, although still rarely used, have a role to play in longitudinal and life course studies of ageing although they normally provide retrospective insights rather than examining changes over time [14, 15]. Data in the form of text from interviews, open-ended questionnaires and written narratives leads to analysis focused on the ways in which people describe their worlds, the meanings they create and how they shape their experiences through narrative [16]. Qualitative sub-studies can add context and richness to longitudinal studies, as well as suggesting new hypotheses to be tested quantitatively. They can also inform person-centred approaches in health and social care [17, 18]. Exploring how older adults understand how their early years have shaped their later life through reflecting on childhoods lived through very different eras than the present day may contribute to more insightful approaches to those who now face the challenges of ageing.

The candidates for early influence in longitudinal studies have normally been readily measurable environmental, social, economic and health factors as well as individual differences. Some aspects of life are not easy to measure and retrospective qualitative studies have the capacity to provide further insights on a range of topics, including how parents convey warmth, respect, security, discipline and values; experiences with siblings; childhood understandings of the social world; educational experiences and so forth. Collecting data on these topics might help us in our search to understand how childhood experience contributes to adulthood and old age, continuities and change within individuals and those aspects of childhood that place individuals at risk for negative outcomes later on and conversely, those which help them become resilient adults and older people.

This research report describes a qualitative life review study of the start in life of 126 members of the LBC1921 near to their 90^th^ birthdays. The study aims to contextualise the early years of the LBC1921 cohort in time and place, as well as analysing participant accounts of their ‘start in life’, particularly in relation to schooling and family support. It explores risk and resilience through ‘start in life’ themes and stories and suggests that some qualitative data can be linked to quantitative data, allowing future investigation of linkages with wider cohort data.

### Historical context

In 1932, when our cohort members sat the Moray House Test as 11 year olds, education in Scotland was universal, free and compulsory for boys and girls until the age of 14. At that time the discipline of psychology was starting to have a strong impact on teacher training and educational methods, supplanting older strict traditions [19]. Muriel Spark’s well-known novel, *The Prime of Miss Jean Brodie*, satirising new and older educational methods, was set in 1930s Edinburgh (and one of our cohort attended the Edinburgh school that the novel was loosely based on at the same time as Muriel Spark). At the time of testing, the LBC1921 cohort would shortly sit their “qualifiers” and if successful, leave primary school to go on to either secondary school or a less academic post-primary school. The final hurdle in the school system was the “higher” examinations, which qualified young people for university or entry to professions. Most children in Scotland at that time left school at leaving age after a couple of years of post-primary education [20].

Those born in 1921 were part of a post-World War One baby boom in Scotland. The shadow of the Great War fell across them. Many fathers would have served and extended families were likely to have suffered losses. In the decade of their birth, life expectancy was only in the mid-50s and infant mortality was around one in ten. By 1932 the country was still in the grip of the Great Depression, which had led to widespread poverty and unemployment [21].

Family life was changing, with extended families less common except in rural areas, family size decreasing and nuclear family structures (households consisting of two parents and their offspring) the norm. Separation and divorce were less common than today. Premature death was still the most common reason for the absence of one parent from the household, affecting perhaps 5 % of families in the UK in the 1930s [22]. Heart disease, lung disease including tuberculosis, and cancer were the big killers of adults. Gender roles were conventional for the times, with mothers usually out of the paid work force and at home raising their families, except in the most difficult of economic circumstances. Homes were often small and overcrowded, with slums in the major cities [21].

## Methods

### Life review questionnaire

An open-ended questionnaire was developed for incorporation into the assessment materials for the Wave 4 of data-gathering with LBC1921 cohort members, contacted as soon as possible after their 90^th^ birthdays. The Life Review Questionnaire was designed with research questions for this cohort in mind, rather than as a standardised tool.

The questionnaire was three A4 pages in length, with eight or nine lines beneath each question, allowing participants to write their responses freely. Its first section, “Start in Life”, the focus of this article, asked participants about family and schooling around the age of 11, which is the age at which they sat the test of mental ability. The introductory text and questions for this section ran as follows:

“We would like you to think back to your schooldays, when you were around 11 years of age, and reflect on how that time in your life prepared you for your adult life.

1. Recalling yourself as school child around 11 years of age, did you enjoy school? Did your schooling bring out the best in you?
2. Recalling yourself as a child around 11 years of age, did your family circumstances bring out the best in you?
3. Overall, do you think that you got off to a good start in life?”

A later section of the questionnaire was designed to elicit lifetime achievements, sources of happiness and challenges, and the final section asked participants to describe how they experience life today. They were also asked to give advice to young people looking to the future.

The questionnaire was mailed to the participants ahead of their Wave 4 assessment and they were asked to complete it in handwriting and bring it to their research clinic appointment. If they were unable to fill out the form themselves, they were invited to dictate responses to a relative or caregiver. Questionnaires were checked at the clinic or home appointment by one of the authors (AP) and any missing data were added at that time.

### Ethics

This study was part of the wider Wave 4 data collection and ethical permission was obtained from the NHS Scotland A Research Ethics Committee (10/MRE00/87). The study complied with the Helsinki Declaration. All participants gave written informed consent for Wave 4 assessment. All participants were able to give their own consent.

### Sample

The 550 original members of the original LBC1921 cohort, all born in 1921, were recruited from Edinburgh and its environs when the members were at an average age 79 years [10, 12]. They were assessed again at mean ages 83, 87, 90 and 92 years. For Wave 4 of data collection, beginning in 2011, 221 remaining participants were identified. Of these, 164 had completed all previous waves of data collection and so were invited for assessment. Twelve participants left the cohort before assessment, either through death, withdrawal or not being contactable. A further 11 were unable to attend, mainly because of poor health or hospitalisation and medical advice prevented contact with one further person. Twenty-four were referred to the study’s Clinical Research Fellow for cognitive assessment since they had a diagnosis of dementia or demonstrated memory problems at the assessment. Some of these did complete the Wave 4 assessment. Altogether, 129 participants completed Wave 4 assessments, undertaken as closely as possible to their 90^th^ birthday.

Only three of the 129 participating in Wave 4 assessments were not able to complete the Life Review Questionnaire. The 126 completed questionnaires were collected in 2011 and 2012 by the study’s staff member (AP) responsible for assessments, conducted either at a scheduled research clinic visit or in people’s homes.

Table 1 provides data on the participants in relation to Moray House intelligence test scores (higher at age 11 than the Scottish population and higher than the larger sized Lothian Birth Cohort at age 79). It also provides data on gender, years in education, social class, marital status, living situation and current health problems.

**Table 1:** LBC1921 Wave 4 Life Review participant data

Measure	*N* (%)	Mean	SD
Moray House Test			
Scottish Population Age 11^a^	87,498	34.5	15.5
LBC1921 (with life review)^b^ Age 11	111	49.4	10.6
LBC1921 (whole sample)^c^ Age 79	550	59.2	10.8
LBC1921 (with life review)^b^ Age 79	125	63.5	8.0
LBC1921 (with life review)^b^ Age 90	120	52.1	13.9
Years in full-time education	129	11.5	2.7
Gender			
Male	58 (46 %)		
Female	68 (54 %)		
Childhood Social class			
Professional	20 (17 %)		
Managerial	33 (27 %)		
Skilled non-manual	45 (37 %)		
Skilled manual	15 (12 %)		
Unskilled	8 (7 %)		
Adult Social Class			
Professional	41 (33 %)		
Managerial	43 (34 %)		
Skilled non-manual	39 (31 %)		
Skilled manual	0 (0 %)		
Unskilled	2 (2 %)		
Marital status			
Married	33 (26 %)		
Single	11 (9 %)		
Divorced	3 (2 %)		
Widowed	78 (62 %)		
Living Alone	84 (67 %)		
Medical problems			
Hypertension	81 (64 %)		
Cardiovascular disease	57 (45 %)		
Diabetes	6 (5 %)		
Cerebrovascular disease	19 (15 %)		
Cancer	27 (21 %)		
Dementia	2 (2 %)		
Thyroid disease	16 (13 %)		
Arthritis	58 (46 %)		

*Note*. The total numbers differ slightly between measures due to (a) missing data on some of the variables and (b) for some variables data for the 129 Wave 4 participants was used rather than for the 126 Life Review Questionnaire completions

^a^Scottish Mental Survey 1932 sample, which included almost a whole Scottish population born in 1921

^b^Subsample of the LBC1921 with completed life review questionnaire at age 90

^c^Whole LBC1921 sample at the study onset at age 79

### Completed questionnaires

Of the questionnaires handed in, 104 (82.5 %) were self-completed, 15 (11.9 %) were filled in by a relative or caregiver and a further seven (5.6 %) individuals did not respond to the query about who filled in the questionnaire. Since questionnaires were checked by the study researcher (AP) at the time of handing in, all questionnaires were complete with only one exception. The missing data were from one male residing in a nursing home who did not complete four of the nine questions.

The handwritten questionnaires were transcribed into Microsoft Word 2007. Legibility was rarely a problem, with only a few phrases or names of places or people proving difficult. Since the focus of the study was not on older people’s literacy, occasional errors of spelling and punctuation were corrected during the transcription processes. Once transcription was complete, responses to each separate question were copied into a Microsoft Excel 2007 spreadsheet, along with other data including LBC1921 cohort identification number, questionnaire number, sex, living circumstances, social class in childhood and adulthood and Moray House Test score data at ages 11, 79, 87 and 90.

Responses varied in length from one to as many as 150 words on individual questions. The mean word count for the whole questionnaire was 250.7 words, with the range from 21 to 821 words, the median length 186 words and the inter quartile range 122 to 297 words. The mean word count for women was 264 compared to 235 for men. The total word count for the transcribed data was 31,584 words.

### Availability of supporting data

Full transcripts of the Life Review Questionnaires are lodged with the Lothian Birth Cohort studies, Centre for Cognitive Ageing and Cognitive Epidemiology, University of Edinburgh. Further information about permission to access the data is available on http://www.lothianbirthcohort.ed.ac.uk

### Qualitative analysis

#### Thematic analysis

The principal qualitative data technique used in the study is thematic analysis, a standard approach well described by Braun & Clarke [23] and defined by them as “a method for identifying, analysing and reporting patterns (themes) within data” (p.79). Open coding, undertaken by the first author, was the thematic analytic technique used for this study, where developing appropriate categories for coding data is an iterative process. A first run through of responses to particular questions led to a draft list of categories and sub-categories describing the data. These categories were defined, set up as columns in MS Excel 2007 and then coded.

Some coding was simple and straightforward, as with the evaluative responses to questions about overall start in life or enjoyment of school, often eliciting yes/no responses which many then elaborated on. As coding proceeded, there were changes to less straightforward categories and sub-categories. Some categories were collapsed, new ones developed or category descriptions altered. Changes sometimes necessitated revisions to coding already completed.

Using thematic analysis in this iterative manner means that the process of reporting on findings cannot be entirely separated from the coding process. As text is drafted and illustrative quotes selected, errors in transcription or coding are discovered and corrected and, on occasion, a new category may emerge or categories grouped together. The advantage of this process of open coding, carried out by one member of the research team rather than several, is that it is capable of more subtlety in interpretation than a fixed coding process because it incorporates processes of constant revision. Braun and Clarke applaud the flexibility of this approach to coding and argue that fixed coding, which does allow for inter-rater reliability checks, only shows whether researchers have coded data in the same way. The open coding approach, on the other hand, values careful interpretation of text, incorporating processes of revision [23].

It should also be noted that thematically coded qualitative data can be reported statistically. In this study, some questions invited direct responses, e.g. ‘Did you enjoy your schooling?’ All participants responded, often with a direct ‘Yes’ or ‘No’ and usually elaborated on their response. Some answered the question indirectly but their responses were coded for positive or negative evaluations of schooling. In contrast, situations and events described in free text responses do not tell us their frequency in the cohort, although the number of mentions may be reported. For example, leaving school early was mentioned by a number of participants, but if there had been direct questioning on this topic, it is likely that more would have reported the experience. Qualitative research, by highlighting situations or events that participants report as having impacted strongly on the course of their lives, can stimulate future quantitative studies that will establish frequencies and correlations.

#### Narrative analysis

Some participants, particularly those who wrote lengthily, constructed a rudimentary life narrative as they made their way through the questionnaire, linking answers across questions to make for narrative flow. This material is mined for its stories of growing up. It is not subjected to any technical forms of narrative analysis, but is used to illustrate key themes in the data, as well as providing richly illustrative material serving to locate the LBC1921 cohort in time and place.

### COREQ guidelines on reporting qualitative research

In order to assure quality, the checklist associated with the COREQ (Consolidated Criteria for Reporting Qualitative Studies) guidelines on reporting qualitative research was consulted during the preparation of this article [24].

## Results and discussion

The ‘Start in Life’ questions focused participants on (but did not limit them to) 1932, when they were age 11, although participant responses ranged across the whole of their growing years. In response to the question “Overall, do you think that you got off to a good start in life?” four-fifths (82.4 %) of responses were positive, including more than a third (37.3) that were emphatically positive. Only one in ten (9.5 %) were negative, including four participants (3.2 %) who were emphatically negative (two of these were the only participants who referred to growing up on remote Scottish islands). Six participants (4.8 %) gave mixed responses.

A preliminary analysis of ‘Start in Life’ responses by gender, intelligence at age 11 and social class of parents showed that 85.3 % of women, compared to 79.3 % of men, believed that they had got off to a good start in life. Looking at the highest fifth and lowest fifth of scorers on the Moray House Test at age 11, 91.3 % of the high scorers said they had a good start in life, compared to 63.6 % of the lowest scorers. Father’s occupational class showed no clear relationship to self-assessment of start in life. All whose fathers were professionals, with the exception of one participant, were positive, as were all bar one of those whose fathers were in unskilled occupations.

### Start in life narratives

Three accounts of “start in life” are presented here, providing typical recollections of schooling and family support and showing how narrative material was often incorporated into questionnaire responses, at least from those who wrote more extensive responses. Names have been changed to preserve anonymity.

*Christina* was the fifth child in a family of nine:

“I had 4 brothers & 4 sisters and although we were poor we had a good family life. My mother was a lovely cook and in those days there was no cooker and all the food was cooked on an open fire. My mother was a very hardworking woman she kept us spotless and was never done washing by hand. In those days there were no washing machines in fact I take after my mother as I wash by hand most days and hang the washing out on the ropes. We lived in a room & kitchen with no hot water & no bathroom. We used to go to the public baths and also go swimming.”

She told us:

“[I] enjoyed school very much. I started school at 5 years old and was always top of the class. There must have been 40 pupils in my class. Our teacher used to take us to Edinburgh Castle & Holyrood palace. I was never off the school as I loved the lessons. I used to get 100 for my writing in those days not like now.”

Christina had to leave school early:

“I left school at 14 much as I would have liked to go on to Higher education. My mother being poor every penny counted in those days so I started work in Crawfords biscuit factory at 10 shillings per week 50p nowadays. I stayed there for 2 years then worked in the Vaults whisky distillers where I learned to bottle whisky (by hand).”

Now widowed and living in sheltered housing, Christina recalled with pleasure how she was able to join the Land Army during World War 2, although tuberculosis put a stop to her outdoor life, leading to a long period of hospitalisation.

*Janice* grew up in the country on a farm with her parents and much older brothers:

“I walked about two miles to school each day on my own. That didn’t seem to bother me. I walked from school to the local hotel where I had a plate of hot soup each day & that was each day till I started at high school. I became dux of school. Once when the road to school was snowbound-I was taken to school in a farm cart!”

She told us that she “never once missed one day at school!” She believed, “looking back on those years”, that her “very happy” family circumstances did bring out the best in her, even though circumstances meant that she could not pursue her chosen career:

“I enjoyed my life living on a farm and having three brothers older than myself. Unfortunately the war broke out and changed the situation. However being on a farm and because of the war–we as a family were lucky that the whole family–and parents–were all lucky and happy to live and work on the farm together. No work–and I would have gone to the gym college in Aberdeen!”

She joined the Young Farmers’ Club and went on to marry a farmer, leading to a “very happy life”.

*John* grew up in a middle class household:

“My parents were excellent as were our family circ [umstances]. There was a family business run by my grandfather & my father & I was born into a large flat in the West End of Glasgow. When I was 10 we moved into a modest bungalow in Bearsden & my brother, my only sibling, was born. My grandfather having in effect retired.... Initially I enjoyed school. Moderately bright, usually in top third of class.

Two years later when I was 12 my father aged 39 died. My grandfather had to come back into the business. The 30s depression was on & the business collapsed. My mother was very capable but with my infant brother to be looked after she had to find employment which she could do from home. Life was financially very difficult for some years.”

John told us how his father’s death and his mother’s later illness had a huge impact on his schooling and early career:

“I did not realize how I was affected by grief but woke up around fourteen with the class steeped in French & Latin & I was lost, but had grasped more than I realised. When I was sixteen Mother had serious illness & I simply left school. Did not sit Highers but got job in an office. As a junior clerk two years later I was very dissatisfied but contracted T.B. & spent 3 years 9 months in hospital. Chance meeting with old B [oys’] B [rigade] officer resulted in work in his office. He was a solicitor and he suggested I qualify. University (Glasgow) entrance exam. English history French & Latin passed two years later–then aged 27.

John went on to become a successful solicitor. It was a difficult start, he says, but he is proud of his own efforts.

These brief stories demonstrate how rich contextual information was gained from some participant accounts of their Scottish childhoods, the narratives giving a different perspective than the thematic analysis presented in the next section.

### Start in life themes

Participants describing schooling and family support during their growing years on the whole reminisced with warmth. Some also told of hardship, loss and other difficulties jeopardising a good start to their lives, but they often told how negative impacts from these situations were ameliorated by family support. The following sections present a thematic analysis of ‘start in life’ material on schooling and family support. Key themes have been italicised and written extracts identified as from male (M) or female (F). Although some responses were more detailed than others, and these have often been utilised in illustrative quotations, the analysis process has accurately weighted positive and negative evaluations.

### Thematic analysis: schooling

#### Enjoying schooling

Around four-fifths of cohort members (78.6 %) enjoyed their schooling, whereas only 10.3 % disliked school. Mixed views were conveyed by 7.9 %, mainly by those who had liked one school or stage of schooling and not another. Two-thirds of participants (65.9 %) expanded on why they had enjoyed or disliked school and this material was thematically analysed.

*Teachers* were the most frequently mentioned reason for enjoying school. Participants recalled teaching of high quality, helpfulness and encouragement from teachers and affection for particular teachers:

“The teachers were excellent & I remember many of them with gratitude.” (F)

“Most teachers were strict but kind and encouraged us to learn.” (M)

Only a few wrote about being afraid of teachers or mistreated by them. One told us:

“I enjoyed school up until one of the teachers used to come up to me and slap me on the right ear. My Mother was told by one of my class friends about what she was doing…it did make me a lot happier when my Mother went to the School and it was all settled.” (M)

*Academic interests and achievements* featured strongly in accounts of enjoying school. Some wrote of their love of learning and “finding out about new things and ideas” (F). Achievements, such as being at the top of the class, becoming dux, winning scholarships or passing examinations, were recalled with pride:

Sports, acting, debating, singing, gymnastics, a school brass band and music were *school activities* enjoyed, with sports the most frequently mentioned.

“I very much enjoyed sport and taking part in athletics and this encouraged me to be competitive and this was well supported by my parents and went on to play tennis and golf.” (F)

One, recalling schoolboy football, said:

“I did enjoy my time at Forfar East. I played for the football team during my last two years at Primary, and was chosen along with R-L-to represent the school in the sprint race at the annual inter-scholastic sports. I won a prize viz. a stamp album in the football dribbling competition at the same sports. (M)

At 90 this man was still going to football matches, accompanied by his son and grandsons.

A few participants referred to not enjoying sports, mainly put down to not having an aptitude for them.

*School friends* were another reason for liking school:

“I did enjoy school & made lots of friends. Two of my school friends, I still kept until they both died at the age of 88.” (F)

#### Did schooling bring out the best?

After being asked whether they had enjoyed school, participants were asked whether schooling brought out the best in them. Around half (49.2 %) specifically responded that that it had brought out the best, though a number of participants who did not answer the question directly but nevertheless described the positive impact of schooling in their written text. Only 12.7 % thought that school *did not bring out the best* in them, although many of those who responded positively to the question went on to elaborate on aspects of schooling that were difficult or discouraging.

*Typically positive responses* to this question included:

“I think we had a very good education & it brought out the best in us.”

“… it did bring out many talents.” (F)

“…school helped me to enjoy life.” (M)

“I think without knowing it, it set me on the right course.” (M)

Some mentioned their academic aptitude, enjoyment of learning or application to schoolwork as reasons for schooling bringing out the best. On the whole, those with positive views of schooling tended to reminisce happily about their experiences and their reminiscences, presented in the section above, on enjoying school, adds to the picture of schooling as bringing out the best in them.

*School difficulties* that were mentioned included not doing well academically because of lack of praise and encouragement, lack of challenge or being expected to conform.

Several participants experienced setbacks due to changing schools and two said that being put forward a year was a problem. Another told of homesickness, having been sent to a boarding school that she did not like as it “concentrated more on producing ‘young ladies’ rather than students” (F).

Some participants made comments on how their own personal qualities or behaviour caused difficulties. One woman, echoing the language of school reports, said that she “should have paid more attention”. A few said they were not very interested in lessons, were more interested in sport, or just had other things to think about:

“I was a bit of a dreamer and just let everything happen, with others helping me when required....Now I realise with a bit of a push in the right way and ambition I could have achieved much more.” (F)

One or two thought that lack of ability kept them from a good start in life:

“I would have it better if I had been quicker on the uptake.” (M)

*Family and personal circumstances* were sometimes mentioned as causing school difficulties. Family influences on schooling are covered in a subsequent section on family support. In terms of personal circumstances, a few participants referred to childhood illnesses or disabilities hampering their schooling:

“I consider that I got off to as good a start as could be expected as I was looked upon as being an ailing child. I never had a complete years attendance at the many schools that I was at and once was absent from school for a full year.” (M)

One woman from the Scottish Islands said that she got off to a poor start in life because schooling was conducted in English:

“I went to school at five years of age, spoke only Gaelic–my teacher was English spoken, so I found school very confusing.”

Leaving school early or attending irregularly, mainly because of difficult family circumstances, stood in the way of educational and career opportunities. *Not completing their education* was the most common regret expressed in relation to schooling, mentioned by 13 participants. Only a couple of participants said that they left early because they had not wanted to stay on.

The most negative comment on school amongst the 126 participants was from a man who told us his father had been an alcoholic and that success in life was entirely due to his own efforts. He said of his education:

“School never did anything for me.” (M).

Overall, participants placed a high value on the education they received, enjoying their schooling, believing that it brought out the best in them and that it gave them a good start in life, especially when circumstances enabled them to make the most of what was offered.

### Thematic analysis: family support

More than three out of four respondents (76.19 %) told us that their family circumstances brought out the best in them, whereas only 13.49 % did not think so. The rest gave neutral or mixed answers. Most of the positive responses were quite emphatic and only one of the 126 respondents was entirely negative about family support.

#### Family life evaluated

What were the key themes in descriptions of supportive families? Around a third of the respondents (34.2 %) referred to a *happy family life* and far more may have done so, judging from the tenor of the responses, had they been specifically asked whether their families were happy. Typical remarks were:

“A marvellous family–I couldn’t have wished for better.” (F))

“My mother and father were lovely people and I often look back and think of all the fun we had.” (F)

Expressions of *unhappiness with family life* were rare. One woman told us:

“…my family was poor & my father was ill & my mother was looking after 7 children. Father came back from war after being gassed and liked to drink.”

She nevertheless expressed thankfulness for her parents.

Around one-third of participants (32.6 %) referred to the ways in which their parents *encouraged schooling*, supporting them to do their best, helping with learning and homework and expanding their horizons:

“My father helped with anything I didn’t understand. He was a very clever man. My Mum played dictionary games, spelling and such.” (F)

References to *lack of interest or neglect* from parents were very rare:

“I was left to do my own thing and spent my time outside playing.” (M)

*Activities* with parents or encouraged by parents were fondly recalled. These included being taken on outings and holidays by the seaside, visiting relatives and participating in cubs and guides, choirs and other church activities and sporting activities.

Many participants commented on how *values and good habits* were emphasized in their families. Household chores, and in some cases farm chores, instilled values around hard physical work, cleanliness and responsibility:

“We were given daily tasks to perform. Such as washing dishes–sweeping floors etc. I was also put in charge of a younger sister & was responsible for her safety. I also had a job delivering newspapers, morning & evening.” (M)

Loyalty to the family, helping each other, considering the needs of others and the learning of Christian ethics were other values emphasised:

“…while in a way I had quite a strict upbringing I soon learned what the difference was between right & wrong.” (F)

“I learned to appreciate what I had & to help & care for people.” (F)

“I learned the family came first, an unwritten rule in Shetland, where large families were the norm.” (F)

Only a few referred to very *strict parents*:

“My mother was the disciplinarian in our house, she set out the rules and regulations, being stubborn I seem to remember going to bed quite often with a sore rear end.” (M)

A number of participants mentioned *siblings* and their position in the family. Brothers and sisters were part of a warmly remembered family life for many. Oldest children sometimes mentioned extra household and caring responsibilities, while youngest children tended to sound carefree.

Family dynamics involving favouritism or unfavourable comparisons with siblings cropped up in very few accounts:

“I had a very talented elder sister and a much younger brother. I wanted to please my parents but found it difficult to fit in.” (F)

Not having siblings was not necessarily regretted:

“I was an only child, and so enjoyed the undivided attention of two loving parents.” (M)

#### Family difficulties

Family difficulties were presented as challenging for parents trying to provide good start in life. *Lack of money* was by far the most frequently mentioned of difficult circumstances, referred to by more than a quarter (28.6 %) of the cohort. Many emphasised how well their parents had managed despite hardship. Being grateful that parents always managed to put food on the table was a repeated theme:

“We were always hard-up…[but] we were always well-fed (Some came to school hungry–we were never like that.)” (M)

Shortage of money meant that a number of LBC1921 participants had to leave school early or could not carry on to the next stage of education, as mentioned earlier. In the accounts of interrupted education, family finances were most frequently mentioned:

“Our parents were encouraging and caring. Unfortunately lack of cash prevented me carrying on at school after 14.” (F)

Being protected and cared for figured in stories of family hardship. One woman, whose father was unemployed, said:

“We knew that money was scarce but that we were held in a loving & secure family relationship.” (F)

Most participants appear to have grown up within nuclear family structures, with two parents and siblings. The four who mentioned *growing up with grandparents* all described their family life with warmth, even though the back story was one of parental difficulty. One man told us:

“I was brought up by my grandmother as my mother had me as an illegitimate child, resulting in my grandfather leaving home & going to Australia so I never had a father or a grandfather but was raised in a Christian home by my grandmother, my mother & my aunt, where I had a life of great happiness.” (M)

Parental separation or divorce appears to have been uncommon, with not a single participant referring to such circumstances. *Parental death* was the most common reason given for living in one parent families, with eight participants mentioning a parent’s death; seven of these had lost their father. Nearly all of these described the family’s finances changing abruptly for the worse. Taking on heavier responsibilities and leaving school early or not being able to continue to university was a common outcome. The one participant whose mother had died did not appear to suffer any economic hardship.

Those who had experienced death of a parent often expressed gratitude for the efforts of their remaining parent to provide for them:

“I had a very happy home life and despite lack of money, my mother did everything in her power to give me a good start.” (F)

*Illness or disability in a parent*, referred to by six participants, was another source of family difficulty:

“My father was disabled due to the 14-18 war and was unable to work. I realised we were quite poor and I was looking to the time I could work and help financially.” (M)

Four participants, all women, told of being kept off school to help in the household because of their mothers’ illness:

“My mother was often ill and I stayed off school to look after my five, soon to be six siblings. I was the eldest girl and expected of me instead of learning.” [F]

LBC1921 accounts of schooldays and family life, focused on age 11 in 1932, were very positive, despite the challenges of that era. Cohort members reported enjoying school, appreciating teachers, making friends and valuing learning opportunities and activities on offer. Families were portrayed, often with great affection, as providing supportive environments that encouraged learning and education. Positive accounts of ‘start in life’ were dominant right across the cohort, although high scorers on the Moray House Test of intelligence at age 11 evaluated their start in life more positively. Women were a little more positive than men and there was no clear differentiation by parental social class.

Among the less positive accounts of ‘start in life’, misfortunes such as poverty, needing to leave school early and parental illness or death were described (and these were often interrelated). Parents (or remaining parents) were often shown as doing their best to protect from the consequences of adversity. The tone of the accounts was resilient, with adversity portrayed as jeopardising life chances, yet with secondary gain in terms of character-building. There were only a few references to personal unhappiness in childhood, arising from lack of confidence, lack of familial warmth, not fitting in at school, or challenges such as ill health or disability. It was notable that none of the participants referred to parental separation and divorce, family violence or parental abuse (apart from smacking, evidently seen as a normal form of punishment at the time).

The response which best gives the flavour of LBC1921 responses to the Start in Life questions was this:

“We were all encouraged to do well and be the best we could be.” (F).

## Conclusions

The reminiscences of education and family life of a 1921-born cohort who survived to age 90 are vividly portrayed in this qualitative study of 126 Scottish childhoods during the late 1920s/early 1930s. The study is especially valuable given the rarity of such a large cohort of well-characterised 90 year olds reflecting on their early lives. Participants were shadowed by World War One and lived through the Great Depression but despite these externally unfavourable circumstances gave largely positive accounts of their start in life.

Research on autobiographical memory has found that older people’s memories of childhood are positively biased, so there may well be a ‘rosy glow’ to the accounts [25-27]. Although participants did not seem reluctant to describe family misfortune, it is possible that personal unhappiness or family conflicts were understated, given the reticence and family loyalty characteristic of this Scottish generation. Moreover, positively framing one’s life history may be one characteristic of the resilient adults of this cohort who have lived a long life. Their positiveness is consistent though, with literature cited in the introduction to the effect that favourable early life circumstances and personal qualities such as higher intelligence boost longevity and healthy ageing.

The less positive aspects of childhood highlighted in their accounts stimulate reflection on how unfavourable environments may put at risk long and healthy lives. The confluence of parental death, poverty and leaving school early are a case in point. The emphasis on parents mitigating adversity and supporting resilience, despite being unable to prevent immediately unfavourable outcomes, suggests lines of research to untangle how both risk and resilience from childhood play out over a lifetime in their impact on health and longevity. Mostly studies of older adults have documented factors that are favourable to risk or resilience, rather than showing how risk is mitigated, although there are exceptions [3]. Also, the lifetime impact of parental separation and divorce, now figuring significantly in children’s lives, would be interesting to compare with parental death, much more common in the childhoods of this cohort, and such comparisons could include research questions around parental strategies for mitigating risk and developing resilience.

This cohort had access to high quality free education which was in the process of becoming modernised, emphasising the child as an individual. Families showed a characteristically Scottish respect for education, even if they were not always able to support their children beyond school leaving age. Was respect for education more pronounced in the families of the more favoured LBC1921 cohort, who in turn demonstrated greater scholastic capacity, as evidenced by their higher than average intelligence scores at age 11? Parents valuing and supporting their children’s education, often seen as the province of well-educated parents, may be a cultural value mitigating childhood risk of poor education regardless of social class. How respect for education as a value inculcated in childhood might contribute to good outcomes later in life is a potentially interesting research topic. It is also interesting that several of the LBC1921 women who achieved well at school and who then became stay-at-home mothers in the post-World War Two era expressed disappointment at not having had a chance to make their mark in the outside world, yet took great pride in having encouraged children and grandchildren to succeed academically. This may have been a strategy for mitigating the risks associated with disappointment around mid-life generativity.

Gender differences were marked in the childhoods and throughout the lives of the cohort studied. However, gender had less impact on accounts of schooling than perhaps could be expected, although reasons for missing school or leaving early were often gender-based. Accounts of family life were also marked by gender, especially in relation to caring duties and preparation for post-school life, but not markedly otherwise.

Topics for discussion and future research arise in relation to this analysis of text provided by the LBC1921 cohort. How reliable are accounts of ‘start in life’ drawn from memories of many years previously? Are the cohort’s accounts characteristic of Scottish childhoods of their time, or do LBC1921’s 90 year olds, having experienced more favourable conditions on average, provide a biased picture? How should we interpret the positivity of descriptions of ‘start in life’: as cognitive bias or as characteristic of survivors? On the other hand, what research questions can we develop from the accounts of childhoods with significant obstacles to a good start?

The Life Review Questionnaire proved a useful tool in eliciting material suitable for qualitative analysis from a cohort of 90 year olds. It could be readily adapted for use with other longitudinal cohorts of older people. If the tool were used with larger cohorts, a recommendation would be to fix categories for coding through examination of an initial sample of material. Fixed rather than open coding would lend itself to measuring inter-rater reliability although this approach would risk some loss of depth in analysis. It could also be interesting to repeat the questionnaire at a different time point with the same cohort, in order to analyse changes in perspective over time and at different ages.

The fact that some of the questions in the Life Review Questionnaire can be coded into values (in particular, the self-assessments of ‘start in life’) means that further work from this Life Review study could involve exploring relationships between ‘start in life’ and some of the extensive cognitive, other psychological, social and health data gathered from the LBC1921 cohort from age 79 onwards.

Key strengths of our Life Review study are that it is sizeable for a qualitative study, it uses a well-described cohort, it vividly locates the early years of that cohort in time and place, it provides insights that could not readily be gained from the mass of quantitative and clinical data available and it generates fresh hypotheses about childhood risk and resilience in relation to ageing that could be further explored in longitudinal studies. Additional limitations include participants being survivors rather than representative of the either the original 550 LBC1921 cohort first wave members or the wider 1932 Scottish population of 11 year olds from which they were drawn. Participants were well favoured compared to their peers, with higher intelligence and parental social class. Their longevity follows on from comparably better health and socioeconomic status over their lifetimes, although they may have been less adventurous than many of their peers, remaining in Scotland rather than immigrating to other countries in search of opportunities. Nor are the participants representative of Scottish 90 year olds today. Our population is from Edinburgh and its environs, a region with a higher socio-economic profile than most other parts of Scotland. Ethnic diversity is not a feature of the study, since all cohort members were living in Scotland in 1932 and so do not reflect the current diversity of the Scottish population.

In conclusion, this qualitative study provides insights into how people in advanced age recall their childhoods–at least, those aspects that they are prepared to share with us -weaving a rich texture of memories and experience into meanings that relate to and probably help create their wellbeing across a lifetime and as older adults.

### Ethics approval and consent to participate

This study was part of the wider Wave 4 data collection and ethical permission was obtained from the NHS Scotland A Research Ethics Committee (10/MRE00/87). The study complied with the Helsinki Declaration. All participants gave written informed consent for Wave 4 assessment. All participants were able to give their own consent.

### Consent for publication

Consent from participants to publish anonymised material has been obtained.

### Availability of data and materials

Full transcripts of the Life Review Questionnaires are lodged with the Lothian Birth Cohort studies, Centre for Cognitive Ageing and Cognitive Epidemiology, University of Edinburgh. Further information about permission to access the data is available on http://www.lothianbirthcohort.ed.ac.uk

## Abbreviations

COREQ: consolidated criteria for reporting qualitative studies
F: female
LBC1921: Lothian birth cohort 1921
LBC1936: Lothian birth cohort 1936
M: male
SES: socioeconomic status
